# Determinants of fertility desire among married or cohabiting individuals in Rakai, Uganda: a cross-sectional study

**DOI:** 10.1186/s12978-016-0272-3

**Published:** 2017-01-10

**Authors:** Joseph K. B. Matovu, Fredrick Makumbi, Rhoda K. Wanyenze, David Serwadda

**Affiliations:** 1 Department of Community Health and Behavioral Sciences, Makerere University School of Public Health, Kampala, Uganda; 2 Department of Epidemiology and Biostatistics, Makerere University School of Public Health, Kampala, Uganda; 3 Family Health Research and Development Center, Makerere University School of Public Health, Kampala, Uganda; 4 Department of Disease Control and Environmental Health, Makerere University School of Public Health, Kampala, Uganda; 5 Makerere University College of Health Sciences, School of Public Health, P.O. Box 7072, Kampala, Uganda

**Keywords:** Fertility desire, Determinants, Married individuals, Rakai, Uganda

## Abstract

**Background:**

Recent trends in fertility rates indicate declines in total fertility rate (TFR) in some sub-Saharan African countries. However, countries such as Uganda continue to have a persistently high TFR partly attributed to strong preferences for large family sizes. We explored the factors that influence fertility desire among married or cohabiting individuals in Rakai, a rural district in southwestern Uganda.

**Methods:**

This cross-sectional study of fertility desire (desire to have another child) was nested in a cluster-randomized demand-creation intervention trial for the promotion of couples’ HIV counseling and testing uptake among married or cohabiting individuals that was conducted in Rakai district between March 1 and April 30, 2015. A total of 1490 married or cohabiting individuals, resident in three study regions with differing background HIV prevalence, were enrolled into the study. Data were collected on socio-demographic, behavioral and fertility-related characteristics. We used a modified Poisson regression model to generate prevalence ratio (PR) as a measure of association for factors that were independently associated with fertility desire. We adjusted for clustering at community level and used STATA version 14.0 for all analyses.

**Results:**

Overall, fertility desire was high (63.1%, *n* = 940); higher in men (69.9%, *n* = 489) than women (57.1%, *n* = 451). More than three-quarters (78.8%, *n* = 1174) had 3+ biological children while slightly more than two-thirds (68.5%, *n* = 1020) reported an ideal family size of 5+ children. Only 30% (*n* = 452) reported that they had attained their desired family size. After adjusting for potential and suspected confounders, the factors that were negatively associated with fertility desire were: age 30–39 (adjusted prevalence ratio [aPR] = 0.82, 95% CI: 0.78, 0.86) and 40+ years (aPR = 0.65, 95% CI: 0.60, 0.71); having six or more biological children (aPR = 0.88, 95% CI: 0.80, 0.97); being HIV-positive (aPR = 0.86, 95% CI: 0.78, 0.95) and ever use of any family planning methods (aPR = 0.93, 95% CI: 0.87, 0.99). Being male (aPR = 1.19, 95% CI: 1.07, 1.33); having primary education (aPR = 1.21, 95% CI: 1.01, 1.44) and having not yet attained the desired family size (aPR = 4.34, 95% CI: 3.50, 5.38) were positively associated with fertility desire.

**Conclusion:**

Having not yet attained one’s desired family size, being male and having primary education were positively associated with fertility desire in this population. Targeting individuals who have not yet attained their desired family size, men and less educated individuals with fertility regulation interventions may help to reduce fertility desire in this population.

## Plain English summary

Worldwide, people are producing fewer and fewer children every day and this has contributed to a reduction in the population sizes in many countries. However, in some countries in sub-Saharan Africa, women still produce more than four children, but more importantly, many women with as many as four children still want to produce more. While the reasons for desiring to have (more) children have been documented in other previous studies, these studies have largely focused on HIV-positive individuals and HIV-discordant couples; but not the general population. In this paper, we explore the reasons for the high desire for children among 1490 married or cohabiting individuals living in Rakai, a rural district in south-western Uganda. Overall, we found that both men and women reported a higher number of living biological children (78.8% had 3 or more children) but on top of this, 68.5% of all individuals interviewed reported that they wanted to have a family size of 5 or more children. Only 30% (452 of 1490) reported that they had attained the number of children that they intended to have. We found that the fact that an individual has not yet attained their desired number was one of the major reasons for the high numbers of children alongside having primary education and being a man. Our findings suggest a need to target those who are still “looking out” for children to attain their desired numbers, individuals with primary education and men in order to regulate fertility levels in this rural population.

## Background

There is evidence to show that total fertility rates (TFR) are declining in the developed and much of the developing world [1, 2], although the trend in some developing countries, including those in sub-Saharan Africa, shows stable or increasing fertility rates [3]. Globally, TFR declined from 4.97 children per woman in 1950–1954 to 2.53 in 2005–2010; in much of the developing world, the changes were even more dramatic over this period, from a TFR of 6.08 to 2.69. However in sub-Saharan Africa, the changes in TFR were much smaller, from 6.53 to 5.39 [1]. The desire for more children, heavily entrenched into strong cultural preferences for large families [4], desire for sons rather than daughters [5] together with low levels of contraception [6], seem to be the driving force for the high fertility rates in sub-Saharan Africa. In support of this observation, a review of reproductive preferences in 60 countries based on data from Demographic and Health Surveys (DHS) conducted between 1998 and 2008 found that, compared to other countries, the number of children desired remained highest in western and middle Africa with an average of 6 children desired during that period [7]. Westoff found that in countries such as Zambia, Tanzania and Uganda, the proportion of women with four or more children who wanted to have another child was more than half of the women interviewed while in Eritrea and Mozambique, between 68 and 78% of women with 4 or more children wanted to have another child [7]. Collectively, these findings suggest that fertility desires remain high in most countries in sub-Saharan Africa, thereby partly explaining the high fertility rates observed in these countries.

Fertility desire can be influenced by a number of factors that operate at the societal and personal/individual levels. At the societal level, fertility desire is largely driven by social cultural pressures and the need to maintain stability of the union [4, 8]. At the personal/individual level, several factors have been associated with fertility desire including age of individuals [9, 10], number of living children [11–13], male sex [10, 14]; enrolment on antiretroviral therapy (among HIV-positive individuals) [14, 15]; partner’s desire to have a child or believing that one’s partner wanted more children [11, 16]; and knowledge of one’s HIV-positive status [17–19]. However, although there is clear documentation of the factors affecting fertility desire in different settings, most studies (with the exception of demographic and health surveys) have been conducted among HIV-positive individuals (and especially among HIV-positive women) and HIV-discordant couples [20]; few studies have explored fertility desire from a general population perspective [18, 19], including among married or cohabiting individuals. This presents a missed opportunity for understanding the factors that influence fertility desire among married or cohabiting individuals who constitute more than half of the adult population in most countries [21]. In this study, we explore the determinants of fertility desire among married or cohabiting individuals enrolled in a population-based study that was conducted in rural Rakai district, southwestern Uganda.

## Methods

### Study design

This was a cross-sectional study that was nested within a cluster randomized demand-creation intervention trial aimed at promoting couples’ HIV counseling and testing (couples’ HCT) uptake among married or cohabiting individuals with no prior couples’ HCT experience in Rakai, Uganda. Details about the cluster-randomized demand-creation intervention trial have been reported elsewhere.

### Study site

The study was conducted in three study regions that are part of the Rakai Community Cohort Study (RCCS) in Rakai district. The RCCS is a population-based cohort that was established in 1994 for a randomized community intervention trial of sexually transmitted infections control for HIV prevention in Rakai district, southwestern Uganda [22]. The cohort consists of 11 study regions; each with approximately 1500 eligible participants (age range: 15–49 years). Previous to data collection, the eleven study regions were grouped into *low* (9.7–11.2%), *medium* (11.4–16.4%) and *high* (20.5–43%) HIV prevalence strata based on HIV prevalence data obtained from the Rakai Community Cohort Study (RCCS) [23]. Each stratum had at least three study regions; one was purposively selected to represent each stratum. The selected study regions were: Buyamba (HIV prevalence = 9.7%, representing low HIV prevalence strata), Katana (HIV prevalence = 12%; representing medium HIV prevalence strata) and Kasensero (HIV prevalence = 43%, representing high HIV prevalence strata).

### Study context

The majority of the population in Rakai district belongs to the Baganda ethnic group [21]. As described by Seeley et al. [24], the descent system among the Baganda is patrilineal with virilogical marriage where women move into the husband’s home upon marriage, in many cases at some distance from their natal home. Similar to other patrilineal societies in Uganda and elsewhere, in Rakai, marriage transfers rights over a female’s sexuality from her family to her new husband. Women are expected to have produce children in their new marriage; failure of which can result into marital instability. Sometimes, women look at child bearing as a form of social protection; believing that this will minimize chances of marital disruption [25]. In Rakai, fatherhood and marriage are part of respectable norms. In a qualitative study conducted to explore how masculine ideals and practices relating to marriage and fertility desires shape young men’s HIV risk, Mathur et al. [26] found that the desire to become a father was instrumental in establishing men’s masculine role and identity within the family in particular and the community in general. Evidence from other studies suggests that having children is an important aspect in men and women’s conceptualization of a long-term relationship [25]. Thus, our study of fertility desire among married or cohabiting individuals was conducted in an area where having children is part and parcel of societal expectations of those who are married or in long-term sexual relationships.

### Study population

The study was conducted among 1490 married or cohabiting individuals (aged 15–49 years) who were resident in each of the three HIV prevalence strata.

### Data collection procedures

Data were collected on socio-demographic (e.g. age, education, religious affiliation), behavioral (e.g. previous receipt of HCT, marital duration) and fertility-related characteristics (e.g. ideal family size, number of living children, whether or not individuals intended to have another child, how long individuals intended to wait before having another child (for those who expressed the desire to have another child), age at first marriage, discussion about how many children a couple should have; and who should decide how many children a couple should have) were collected from all married or cohabiting individuals. All respondents had their data linked to the pre-existing RCCS HIV database to ascertain HIV status (where HIV status information was available) but no fresh blood samples were collected for HIV serology. Data collection took place between March 1 and April 30, 2015.

### Measurement variables

The primary outcome of the study was fertility desire. This was defined as the need to have another child in future as expressed by married or cohabiting individuals at the time of interview. Initially, respondents were asked if they had any biological children to which all individuals responded in the affirmative. These individuals were then asked if they desired to have another child in the future. Those that desired to have another child in the future were asked about how far in the future (months/years) they wanted to wait to have another child, and the period of waiting was categorized as <1 year, 1–2 years, 3–4 years or 5 or more (5+) years. The question on whether or not individuals desired to have another child in the future was asked to all respondents regardless of whether the female partner reported that she was currently pregnant or the male partner reported that his [female] partner was currently pregnant. To determine fertility desire, we combined desire for children as expressed by those who were/whose partners were not currently pregnant and those who were/whose partners were currently pregnant and divided the sum by the total number of respondents. Thus, the term ‘fertility desire’ as used in this paper refers to desire for another child in the future.

We used the term ‘ideal family size’ to refer to the number of children that men and women would have liked to produce if they could go back in their reproductive life and start all over again –after responding to the question: “*If you could go back to the time you did not have any children and could choose exactly the number of children to have in your whole life, how many would that be?*” This question was adopted from the Uganda Demographic and Health Survey Questionnaire. We defined ‘number of biological children’ as the number of living biological children that the respondents had at the time of interview. We used the term ‘ever use of family planning’ to refer to use of *any* family planning method (including modern and traditional methods). We did not have a specific question on ever or current use of modern methods; so, this is not captured in this analysis. Finally, we created a variable, “achieved desired family size” by subtracting the ideal number of children from the number of biological children that individuals had. This variable was necessary to ascertain the extent to which fertility desire changes when the desired family size has been attained as opposed to when the desired family size has not yet been attained.

### Data analysis

We conducted descriptive analysis to assess the characteristics of married or cohabiting individuals enrolled into the study (stratified by sex) and used inferential statistics to ascertain the determinants of fertility desire among married or cohabiting individuals resident in the three study regions. At the bivariate analysis, we assessed the association between fertility desire and each of the independent correlates (socio-demographic, behavioral and fertility-related characteristics) and all variables with a *p*-value <0.05 (i.e. sex, education, age-group, number of biological children, ideal family size, pregnancy status, age at first marriage, whether or not individuals attained their desired family size, and ever use of family planning methods) and suspected confounders (i.e. HCT receipt status; marital duration; HIV status at baseline) were considered for the multivariable model. The suspected confounders were selected based on evidence from previous studies [18, 19, 27]. We used a modified Poisson regression model with a log link to assess the factors that were independently associated with fertility desire after adjusting for clustering at community level. A *p*-value of less than 0.05 was considered significant at the multivariable analysis level. Analysis was conducted using STATA statistical software version 14.0.

### Ethical considerations

The study was approved by the Higher Degrees, Research and Ethical Committee of Makerere University School of Public Health and the Uganda National Council for Science and Technology.

## Results

### Respondent characteristics

Overall, 1490 respondents were interviewed for this study; 53% (*n* = 790) of whom were women (Table 1). Sixty four per cent of men and 82% of women were aged between 19 and 39 years [mean age: 36.9 years (SD: ±6.6) for men and 32.7 years (SD: ±6.9) for women]. Women were married at a younger age than men: 68.4% (*n* = 540) of women were married by age 18 years compared to 13.7% (*n* = 96) of men who were married at that age. Eighty four per cent of respondents had been married for five or more years. Slightly more than two-thirds of respondents (67.9%, *n* = 1012) had primary education; 26.2% (*n* = 390) had post-primary education while the remaining 5.9% (*n* = 88) had no formal education at all. Forty two per cent of all respondents reported that they tested for HIV within the 12 months preceding the study; most as individuals (31.5%, *n* = 470). Nearly six per cent of men and women were living with HIV. Majority of the participants ascribed to the Catholic faith (56.4%); with similar proportions of men (56.3%) and women (56.6%) ascribing to this faith.

**Table 1:** Socio-demographic characteristics of married or cohabiting individuals stratified by sex

Characteristic	Female	Male	Total
	*N* = 790(%)	*N* = 700(%)	*N* = 1490(%)
Age group			
19–29	276 (34.9)	120 (17.2)	396 (26.6)
30–39	373 (47.2)	325 (46.4)	698 (46.8)
40+	141 (17.9)	255 (36.4)	396 (26.6)
Education level			
None	64 (8.1)	24 (3.4)	88 (5.9)
Primary	520 (65.8)	492 (70.3)	1012 (67.9)
Post-primary	206 (26.1)	184 (26.3)	390 (26.2)
Age at first marriage			
≤ 18	540 (68.4)	96 (13.7)	636 (42.7)
19–24	212 (26.8)	380 (54.3)	592 (39.7)
25+	38 (4.8)	224 (32.0)	262 (17.6)
Marital duration			
≤ 2 years	33 (4.2)	58 (8.3)	91 (6.1)
3–4 years	65 (8.2)	88 (12.6)	153 (10.3)
5+ years	692 (87.6)	554 (79.1)	1246 (83.6)
HCT receipt status (past 12 months)			
No HCT	433 (54.8)	429 (61.3)	862 (57.9)
Individual HCT	283 (35.8)	187 (26.7)	470 (31.5)
Couple HCT	74 (9.4)	84 (12.0)	158 (10.6)
Number of biological children			
1–2	170 (21.5)	146 (20.9)	316 (21.2)
3–5	399 (50.5)	307 (43.9)	706 (47.4)
6+	221 (28.0)	247 (35.2)	468 (31.4)
Ideal family size			
0–4	298 (37.7)	172 (24.5)	470 (31.5)
5–6	324 (41.0)	251 (35.9)	575 (38.6)
7+	168 (21.3)	277 (39.6)	445 (29.9)
HIV status			
HIV-negative	688 (87.1)	606 (86.6)	1294 (86.8)
HIV-positive	47 (5.9)	41 (5.9)	88 (5.9)
Unknown HIV status	55 (7.0)	53 (7.5)	108 (7.3)
Religion			
Catholic	447 (56.6)	394 (56.3)	841 (56.4)
Protestant	123 (15.6)	122 (17.4)	245 (16.4)
Saved/Pentecostal	57 (7.2)	45 (6.4)	102 (6.9)
Muslim	163 (20.6)	139 (19.9)	302 (20.3)
Ever use of family planning^a^			
Yes	662 (83.8)	236 (33.7)	898 (60.3)
No	128 (16.2)	464 (66.3)	592 (39.7)
Achieved desired family size			
Yes	263 (33.3)	189 (27.0)	452 (30.3)
No	527 (66.7)	511 (73.0)	1028 (69.7)
Study region			
Buyamba (Low HIV prevalence: 9.7%)	290 (36.7)	262 (37.4)	552 (37.1)
Katana (Medium HIV prevalence: 12%)	302 (38.2)	239 (34.2)	541 (36.3)
Kasensero (High HIV prevalence: 43%)	198 (25.1)	199 (28.4)	397 (26.6)

^a^Ever use of family planning was used in the generic sense, incorporating use of any family planning methods

Nearly half of the respondents (47.4%, *n* = 706) had between 3 and 5 biological children with a slightly higher proportion of women reporting that they had 3–5 children (50.5%, *n* = 399) than men (43.9%, *n* = 307). However, men were 1.4 times (OR = 1.40, 95% CI: 1.12, 1.76) more likely to report having six or more children than women. On the other hand, nearly 40% (*n* = 575) reported an ideal family size of between 5 and 6 children; with a higher proportion of women (41.0%, *n* = 324) than men (35.9%, *n* = 251) reporting this number. However, the proportion of men who reported an ideal family size of 7 or more children (7+) was higher in men (39.6%, *n* = 277) than women (21.3%, *n* = 168). We found that only 30.3% (*n* = 452) of the individuals interviewed had achieved their desired family size, with slightly more than one-third of women (33.3%, *n* = 263) and 27% of men (*n* = 189) reporting that they had attained their desired family size. Ever use of family planning was high with 60.3% reporting that they had ever used any method of family planning. As expected, a higher proportion of women (83.8%, *n* = 662) reported that they had ever used any family planning method than men (33.7%, *n* = 236).

### Fertility-related characteristics

**Fig. 1:**
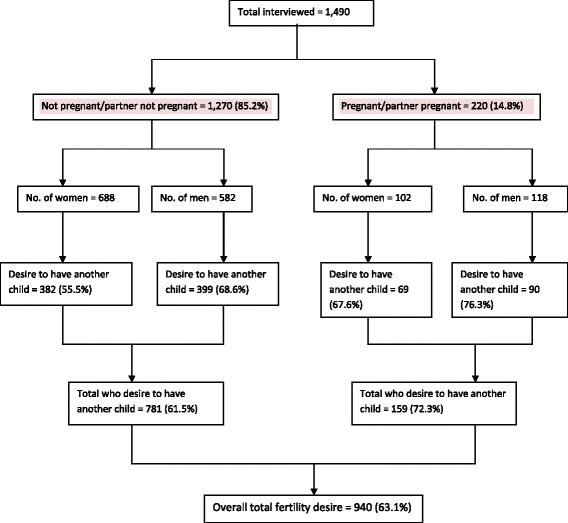
Fertility desire by pregnancy status

Figure 1 shows fertility desire stratified by pregnancy status. Of the 1490 respondents, 85.2% (*n* = 1270) reported that they were not pregnant (women) or had non-pregnant partners (men) while 220 (14.8%) reported that they were currently pregnant (women) or had pregnant partners (men). Overall, fertility desire was significantly higher among women who were pregnant/male partners of pregnant women than women who were not pregnant/male partners of non-pregnant women (72.3% vs. 61.5%, *P* = 0.002). However, when pregnancy status was examined by sex, men had a higher fertility desire than women. For instance, among non-pregnant women/men with non-pregnant partners, 68.6% (*n* = 399) of men wanted to have another child compared to 55.5% (*n* = 382) of women (*P* < 0.0001). Similarly, among pregnant women/men with pregnant partners, 76.3% (*n* = 90) of men wanted to have another child compared to 67.6% (*n* = 69) among women (*P* = 0.15).

Overall, 63.1% (*n* = 940) of both men and women desired to have another child in future; this desire was higher in men (69.9%, *n* = 489) than women (57.1%, *n* = 451). Majority of those who wanted to have another child (42.2%, *n* = 396) wanted to wait for 1–2 years; 29% (*n* = 277) wanted to wait for 3–4 years; 14.7% (*n* = 138) wanted to wait for five or more years, while 13.7% (*n* = 129) wanted to wait for less than one year to have another child (Table 2). When respondents were asked about who should decide the number of children that they should have, 42.9% (*n* = 640) reported that men should be the ones to decide; 26.8% (*n* = 399) reported that it should be the women to decide, while 30.8% (*n* = 451) reported that both men and women should take part in making this decision. However, when these results were stratified by sex, two out of every five women (43%, *n* = 340) reported that it should be the women to decide while more than half of the men (53.6%, *n* = 375) reported that it should be the men to decide the number of children that a couple should have.

**Table 2:** Fertility-related characteristics of married or cohabiting individuals, stratified by sex

Characteristic	Female	Male	Total
	*n* (%)	*n* (%)	*n* (%)
Would you like to have another child in future			
No	339 (42.9)	211 (30.1)	550 (36.9)
Yes	451 (57.1)	489 (69.9)	940 (63.1)
Total	790 (100.0)	700 (100.0)	1490 (100.0)
Of those that would like to have another child, how long would you like to wait?^a^			
< 1 year	75 (16.6)	54 (11.0)	129 (13.7)
1–2 years	158 (35.0)	238 (48.7)	396 (42.1)
3–4 years	139 (30.8)	138 (28.2)	277 (29.5)
5+ years	79 (17.6)	59 (12.1)	138 (14.7)
Total	451 (100.0)	489 (100.0)	940 (100.0)
Who should decide number of children?			
Men	265 (33.6)	375 (53.6)	640 (42.9)
Women	340 (43.0)	59 (8.4)	399 (26.8)
Men and women	185 (23.4)	266 (38.0)	451 (30.3)
Total	790 (100.0)	700 (100.0)	1490 (100.0)
Did you discuss fertility issues with anyone?			
No	213 (27.0)	291 (41.6)	504 (33.8)
Yes	577 (73.0)	409 (58.4)	986 (66.2)
Total	790 (100.0)	700 (100.0)	1490 (100.0)
Did you discuss fertility issues with your partner?^b^			
No	32 (5.5)	51 (12.5)	83 (8.4)
Yes	545 (94.5)	358 (87.5)	903 (91.6)
Total	577 (100.0)	409 (100.0)	986 (100.0)
Did you reach consensus when you discussed fertility issues with your partner?^c^			
Yes, reached consensus	386 (70.8)	326 (91.1)	712 (78.9)
Yes, but wife wanted more	21 (3.9)	15 (4.2)	36 (4.0)
Yes, but husband wanted more	89 (16.3)	09 (2.5)	98 (10.8)
No, we did not reach consensus	49 (9.0)	08 (2.2)	57 (6.3)
Total	545 (100.0)	358 (100.0)	903 (100.0)

^a^Expressed as a proportion of those who wanted to have another child

^b^Expressed from those who reported discussing fertility issues with anyone

^c^Estimated from those who reported that they discussed fertility issues with partner

Two thirds (66.2%, *n* = 986) of the respondents reported that they had ever discussed fertility-related issues with anyone; with a higher proportion of women (73%, *n* = 577) than men (58.4%, *n* = 409) reporting that they did so. Majority of those who had ever discussed fertility issues with anyone reported that they discussed with their primary partner (91.6%, *n* = 903). Women (94.5%, *n* = 545) were significantly more likely to report discussing fertility issues with their primary partners than men (87.5%, *n* = 358; *P* = 0.0001). When asked about the outcome of the discussion, 78.9% (*n* = 712) reported that they reached consensus on the number of children they wanted to have; with a higher proportion of men (91.1%, *n* = 326) than women (70.8%, *n* = 386) reporting that they reached consensus on the fertility issues discussed.

### Effect of HIV prevalence strata on fertility desire

We found that fertility desire was significantly lower among individuals resident in the low HIV prevalence strata (60%, *n* = 331) compared to those resident in the medium (62.3%, *n* = 337) and high HIV prevalence strata (68.5%, *n* = 272); *p* = 0.02. However, when these results were adjusted for potential confounders, we found no significant difference in fertility desire between individuals in the low and medium HIV prevalence strata (adjusted Prevalence Ratio [aPR] = 1.01, 95% Confidence Interval (95% CI): 0.96, 1.06) and between individuals in the low and high HIV prevalence strata (aPR = 0.98, 95% CI: 0.94, 1.02) (data not shown).

### Determinants of fertility desire

At the bivariate analysis, the factors that were negatively associated with fertility desire were age 30–39 and 40+ years, having six or more children, having an ideal family size of 5–8 or 9+ children, marital duration of 5 or more years, being currently pregnant and ever use of family planning methods. On the other hand, the factors that were positively associated with fertility desire were being male, having primary or post-primary education, age at first marriage being 19–24 or 25+ years, having unknown HIV status and not having achieved one’s family size. After adjusting for potential and suspected confounders (Table 3), the factors that were negatively associated with fertility desire were: age 30–39 (aPR = 0.82, 95% CI: 0.78, 0.86) and 40+ years (aPR = 0.65, 95% CI: 0.60, 0.71); having six or more children (aPR = 0.88, 95% CI: 0.80, 0.97), being HIV-positive (aPR = 0.86, 95% CI: 0.78, 0.95) and ever use of family planning methods (aPR = 0.93, 95% CI: 0.87, 0.99). On the other hand, the factors that were positively associated with fertility desire were: not yet having achieved one’s family size (aPR = 4.34, 95% CI: 3.50, 5.38); being male (aPR = 1.20, 95% CI: 1.07, 1.33) and having primary education (aPR = 1.21, 95% CI: 1.01, 1.44) compared to no formal education. There was no significant difference in fertility desire between those who did not receive HCT in the past 12 months and those who received HCT alone (aPR = 0.97, 95% CI: 0.87, 1.08) or together with their partners (aPR = 1.09, 95% CI: 0.98, 1.41). Also, there was no significant difference in fertility desire between HIV-negative individuals and individuals with unknown HIV status (aPR = 1.10, 95% CI: 0.97, 1.23).

**Table 3:** Unadjusted and adjusted prevalence ratios of fertility desire among married or cohabiting individuals in Rakai, Uganda

Variable	Unadjusted prevalence ratio and 95% Confidence Intervals	Adjusted prevalence ratio and 95% Confidence Intervals^a^
Sex		
Female	1.00	1.00
Male	1.22 (1.13, 1.32)	1.19 (1.07, 1.33)
Education		
None	1.00	1.00
Primary	1.62 (1.28, 2.05)	1.21 (1.01, 1.44)
Post-primary	1.62 (1.29, 2.05)	1.18 (0.98, 1.41)
HCT receipt in past 12 months		
No HCT	1.00	1.00
Individual HCT	0.95 (0.83, 1.08)	0.97 (0.87, 1.08)
Couples’ HCT	1.08 (0.96, 1.23)	1.09 (0.98, 1.41)
Age group		
19–29	1.00	1.00
30–39	0.72 (0.67, 0.78)	0.82 (0.78, 0.86)
40+	0.47 (0.40, 0.54)	0.65 (0.60, 0.71)
Number of biological children		
1–2	1.00	1.00
3–5	0.34 (0.06, 1.81)	1.04 (0.96, 1.12)
6+	0.08 (0.02, 0.29)	0.88 (0.80, 0.97)
Ideal family size		
0–4	1.0	1.00
5–8	1.80 (0.74, 0.87)	0.94 (0.86, 1.02)
9+	0.41 (0.35, 0.47)	0.96 (0.87, 1.06)
Marital duration		
1–2 years	1.00	1.00
3–4 years	1.10 (0.93, 1.31)	1.02 (0.90, 1.15)
5+ years	0.79 (0.67, 0.92)	1.03 (0.92, 1.14)
Age at first marriage		
≤ 18	1.00	1.00
19–24	1.14 (1.05, 1.25)	0.98 (0.91, 1.07)
25+	1.20 (1.05, 1.38)	1.05 (0.98, 1.13)
Pregnancy status		
No, not pregnant	1.00	1.00
Yes, pregnant	0.85 (0.77, 0.94)	1.06 (0.99, 1.13)
HIV status at baseline		
Negative	1.00	1.00
Positive	0.94 (0.77,1.44)	0.86 (0.78, 0.95)
Unknown HIV status	1.12 (1.02, 1.24)	1.10 (0.97, 1.23)
Achieved desired family size		
Yes	1.00	1.00
No	5.09 (4.10, 6.32)	4.34 (3.50, 5.38)
Ever used family planning		
No	1.00	1.00
Yes	0.84 (0.74, 0.95)	0.93 (0.87, 0.99)

^a^Adjusted for clustering at community level

## Discussion

Our study of fertility desire among married or cohabiting individuals in Rakai, Uganda, found that six in every ten married or cohabiting individuals desired to have another child. The observed fertility desire is higher than what has been reported in most studies [11–13] but closer to fertility desires previously reported in Rakai, Uganda [28]. The higher fertility desire in this population could be partly explained by the fact that majority of the respondents (64% of men and 82% of women) were below the age of 40 years (and were therefore still in the prime of their reproductive years) and partly due to the fact that majority of the women were married at a younger age (68.4% by 18 years), thereby increasing the reproductive life span [29].

The factors that were significantly associated with an increased desire to have another child were: being male, having primary education and having not yet achieved the desired family size. Ever use of *any* family planning methods, being HIV-positive, having six or more children, and age-group 30–39 or 40+ years were associated with less desire for children in the future. The finding that being male was positively associated with fertility desire is in direct consonance with previous findings on this subject [10, 14] and is consistent with the belief, expressed by 43% of the respondents, that men should be the ones to decide the number of children that a couple should have. Studies show that men tend to desire to have large families [29–31] which supports the finding that men were significantly more likely to desire to have more children than women. However, since having a husband whose desired number of children is smaller than the wife’s can increase the odds of having a small family size [32], our findings suggest a need to engage men as an important target group in fertility regulation interventions [30].

The finding that individuals who had not yet attained their desired family size had a higher desire for children compared to their counterparts was not surprising given that there is already evidence showing that the ideal number of children that individuals would like to have is a significant predictor of fertility desire [27]. The relationship between ideal and desired family sizes can be a straight one: if the ideal number of children that people wanted to have was high, it follows that fertility desire can increase to match with the high ideal numbers of children desired [27]. Thus, our findings have implications for fertility control programs and suggest a need to target individuals who have not yet attained their desired family sizes as well as non-users of family planning in order to ensure that those who do not desire any more children do not get unwanted pregnancies.

Related to ideal family size is the actual number of biological children that men and women have. Studies show a decreasing desire for children as the actual number of biological children increases [11, 14, 27]. For instance, findings from the most recent Uganda Demographic and Health Survey [27] show that the proportion of women who would like to have another child soon decreased from 78.9% among those with no children to 8.6% among those with four children and 3.4% among those with six or more children. Similarly, the proportion of men who would like to have another child soon decreased from 77.5% among men with no children to 15.8% among those with four children and 9.6% among those with six or more children. We found that having 6 or more children was significantly associated with less likelihood of desiring to have another child. However, there was no significant difference in fertility desire between those who had 3–5 when compared to those with 1–2 children, possibly explained by the fact that, in this population, 68.5% of the respondents desired to have a family size of 5 or more children. In order to regulate population growth, our findings suggest a need for programs that promote contraceptive use to target individuals with fewer children who are also likely to be young and, by implication, to have high fertility desire.

Our finding that HIV-positive individuals were less likely to desire more children was consistent with other studies that found low fertility desires among HIV-positive individuals [17, 18, 28]. However, our findings are in sharp contrast with findings from other studies that have found increased fertility desire among HIV-positive individuals, particularly among those enrolled on antiretroviral therapy [15, 19, 33]. These contrasting findings suggest that other factors beyond a person’s antiretroviral therapy enrolment status – a partner’s desire to have a child or the belief that a partner would like to have a child [16], number of living children that men and women have [13], age [10], being male [14] and lifetime history of not having a live birth [4] – operate at a more proximal level to influence fertility desire among HIV-positive individuals. Thus, fertility regulation interventions targeting HIV-positive individuals should integrate components that focus on some of these proximal determinants to assist people living with HIV to make informed, rationale reproductive health decisions.

We found that having primary or post-primary education was positively associated with fertility desire at the bivariate analysis, suggesting that higher levels of education were associated with higher levels of fertility desire. These findings contrast previous findings on this subject that higher education is associated with lower fertility desire [34, 35]. When these results were adjusted for potential confounders, having primary rather than post-primary education remained positively associated with fertility desire. We do not know why individuals with primary education were significantly more likely to have higher fertility desire than those with no formal education. It is also not particularly clear why fertility desire did not differ between those with no formal education and those with post-primary education. Further research is warranted to explore the relationship between level of education and fertility desire in this population.

This study had several limitations. Although fertility desire can be influenced by ART coverage levels, and there is already evidence that ART coverage is increasing in Rakai [15, 28], our study did not collect data on ART coverage; so, we did not adjust for the effect of ART availability on fertility desire in the final model. However, since our population included both HIV-positive, HIV-negative and individuals with unknown HIV status, our findings can still provide a general reflection of the fertility desire at population level. The other limitation was that we did not collect any fresh blood samples to determine HIV status but relied on HIV status data that were already available within the RCCS HIV results database. As noted in Table 1, nearly six out of every ten respondents interviewed did not participate in HCT in the 12 months preceding the study, suggesting that the available HIV status data might not reflect the exact HIV status at the time of the study due to likely HIV sero-conversion among previously HIV-negative respondents. Finally, our inability to collect data on current use of modern family planning methods was a key limitation, since these methods control fertility levels. However, given the low levels of modern contraceptive use in Uganda [27], and the fact that contraception tends to reduce with increased fertility desire [36], we can safely conclude that the fertility desire observed in our study population reflect a more realistic picture of the fertility levels in this rural population.

## Conclusion

Our study of fertility desire among married or cohabiting individuals in Rakai, Uganda, found that six in every ten married or cohabiting individuals desired to have another child. Having not yet attained one’s desired family size, being male and having primary level of education were positively associated with fertility desire in this population. However, being HIV-positive, having six or more children, age-group 30–39 and 40+ years and ever use of any family planning methods were associated with reduced fertility desire in this population. Our findings suggest a need to target individuals who have not yet attained their desired family sizes, men and less educated individuals with fertility regulation interventions, including family planning, in order to control fertility levels in this population.

## Abbreviations

aPR: Adjusted prevalence ratio
ART: Antiretroviral therapy
DHS: Demographic and health survey
HCT: HIV counseling and testing
HIV: Human immunodeficiency virus
PR: Prevalence ratio
RCCS: Rakai Community Cohort Study
SD: Standard Deviation
STI: Sexually transmitted infection

## References

[1] United Nations, Department of Economic and Social Affairs, Population Division. Fertility levels and trends as assessed in the 2012 revision of World Population Prospects. United Nations; 2013. http://www.un.org/en/development/desa/population/publications/pdf/fertility/Fertility-levels-and-trends_WPP2012.pdf. Accessed 7 July 2016.

[2] Shapiro D, Gebreselassie T (2008). Fertility transition in sub-Saharan Africa: falling and stalling. Afr Popul Stud.

[3] Lesthaeghe R (2014). The fertility transition in sub-Saharan Africa into the 21st Century: Population Studies Center Research Report 14–823.

[4] Dyer SJ, Abrahams N, Hoffman M, van der Spuy ZM (2002). ‘Men leave me as I cannot have children’: women’s experiences with involuntary childlessness. Hum Reprod.

[5] Chauduri S (2012). The desire for sons and excess fertility: a household-level analysis of parity progression in India. Int Perspect Sex Reprod Health.

[6] Creanga AA, Gillespie D, Karklins S, Tsui AO (2011). Low use of contraception among poor women in Africa: an equity issue. Bull World Health Organ.

[7] Westoff CF (2010). Desired number of children: 2000–2008. DHS Comparative Reports No. 25.

[8] Ngure K, Baeten JM, Mugo N, Curran K, Vusha S, Heffron R, Celum C, Shell-Duncan B (2014). My intention was a child but I was very afraid: fertility intentions and HIV risk perceptions among HIV-serodiscordant couples experiencing pregnancy in Kenya. AIDS Care.

[9] Asfaw HM, Gashe FE (2014). Fertility intentions among HIV positive women aged 18–49 years in Addis Ababa Ethiopia: a cross sectional study. Reprod Health.

[10] Wagner GJ, Wanyenze R. Fertility desires and intentions and the relationship to consistent condom use and provider communication regarding childbearing among HIV clients in Uganda. ISRN Infect Dis. 2013;2013:Article ID 478192. 10.5402/2013/478192 PMC4219363 25379322

[11] Gutin SA, Namusoke F, Shade SB, Mirembe F (2014). Fertility desires and intentions among HIV-positive women during the post-natal period in Uganda. Afr J Reprod Health.

[12] Negash S, Yusuf L, Tefera M (2013). Fertility desires predictors among people living with HIV/AIDS at art care centers of two teaching hospitals in Addis Ababa. Ethiop Med J.

[13] Kawale P, Mindry D, Stramotas S, Chilikoh P, Phoya A, Henry K, Elashoff D, Jansen P, Hoffman R (2014). Factors associated with desire for children among HIV-infected women and men: a quantitative and qualitative analysis from Malawi and implications for the delivery of safer conception counseling. AIDS Care.

[14] Kipp W, Heys J, Jhangri GS, Alibhai A, Rubaale T (2011). Impact of antiretroviral therapy on fertility desires among HIV-infected persons in rural Uganda. Reprod Health.

[15] Litwin LE, Makumbi FE, Gray R, Wawer M, Kigozi G, Kagaayi J, Nakigozi G, Lutalo T, Serwada D, Brahmbhatt H (2015). Impact of availability and use of ART/PMTCT services on fertility desires of previously pregnant women in Rakai, Uganda: a retrospective cohort study. J Acquir Immune Defic Syndr (1999).

[16] Beyeza-Kashesya J, Ekstrom AM, Kaharuza F, Mirembe F, Neema S, Kulane A (2010). My partner wants a child: a cross-sectional study of the determinants of the desire for children among mutually disclosed sero-discordant couples receiving care in Uganda. BMC Public Health.

[17] Yeatman SE (2009). The impact of HIV status and perceived status on fertility desires in rural Malawi. AIDS Behav.

[18] Taulo F, Berry M, Tsui A, Makanani B, Kafulafula G, Li Q, Nkhoma C, Kumwenda JJ, Kumwenda N, Taha TE (2009). Fertility intentions of HIV-1 infected and uninfected women in Malawi: a longitudinal study. AIDS Behav.

[19] Dube AL, Baschieri A, Cleland J, Floyd S, Molesworth A, Parrott F, French N, Glynn JR (2012). Fertility intentions and use of contraception among monogamous couples in northern Malawi in the context of HIV testing: a cross-sectional analysis. PLoS One.

[20] Berhan Y, Berhan A (2013). Meta-analyses of fertility desires of people living with HIV. BMC Public Health.

[21] Uganda Bureau of Statistics (UBOS) (2016). The National Population and Housing Census 2014 – Main Report.

[22] Wawer MJ, Gray RH, Sewankambo NK, Serwadda D, Paxton L, Berkley S, McNairn D, Wabwire-Mangen F, Li C, Nalugoda F (1998). A randomized, community trial of intensive sexually transmitted disease control for AIDS prevention, Rakai, Uganda. AIDS.

[23] Grabowski MK, Lessler J, Redd AD, Kagaayi J, Laeyendecker O, Ndyanabo A, Nelson MI, Cummings DA, Bwanika JB, Mueller AC (2014). The role of viral introductions in sustaining community-based HIV epidemics in rural Uganda: evidence from spatial clustering, phylogenetics, and egocentric transmission models. PLoS Med.

[24] Seeley J, Kajura E, Bachengana C, Okongo M, Wagner U, Mulder D (1993). The extended family and support for people with AIDS in a rural population in south west Uganda: a safety net with holes?. AIDS Care.

[25] Agol D, Bukenya D, Seeley J, Kabunga E, Katahoire A (2014). Marriage, intimacy and risk of HIV infection in south west Uganda. Afr J Reprod Health.

[26] Mathur S, Higgins JA, Thummalachetty N, Rasmussen M, Kelley L, Nakyanjo N, Nalugoda F, Santelli JS (2016). Fatherhood, marriage and HIV risk among young men in rural Uganda. Cult Health Sex.

[27] Uganda Bureau of Statistics (UBOS), ICF International Inc. Uganda Demographic and Health Survey. Kampala, Uganda: UBOS and Calverton, Maryland: ICF International Inc.; 2012. www.ubos.org/onlinefiles/uploads/ubos/UDHS/UDHS2011.pdf. Accessed 7 July 2016.

[28] Mathur S, Zhong X, Lutalo T, Wunder K, Wei Y, Wawer M, Santelli JS. ART availability and fertility desire: evidence from a population-based cohort in Rakai, Uganda from 2001–2011. San Diego: Paper presented at the 2015 Population Association of America Meeting; 2015.

[29] Garenne M. Trends in marriage and contraception in sub-Saharan Africa: a longitudinal perspective on factors of fertility decline. DHS Analytical Studies No. 42. In. Rockville: ICF International; 2014. http://dhsprogram.com/pubs/pdf/AS42/AS42.pdf. Accessed 4 July 2016.

[30] Ratcliffe AA, Hill AC, Walraven G (2001). The ignored role of men in fertility awareness and regulation in Africa. Afr J Reprod Health.

[31] Ratcliffe AA, Hill AC, Walraven G (2000). Separate lives, different interests: male and female reproduction in The Gambia. Bull World Health Organ.

[32] Carvalho A, Wong LR. Are Brazilian women having fewer children than they desire? A Comparison between 1996 and 2006. San Diego: Paper presented at the 2015 Population Association of America Meeting; 2015.

[33] Bonnenfant YT, Hindin MJ, Gillespie D (2012). HIV diagnosis and fertility intentions among couple VCT clients in Ethiopia. AIDS Care.

[34] Jiang L, Hardee K (2014). Women’s education, family planning or both? Application of multistate demographic projections in India. Int J Popul Res.

[35] Martin T (1995). Women’s education and fertility: results from 26 demographic and health surveys. Stud Fam Plann.

[36] OlaOlorun F, Seme A, Otupiri E, Ogunjuyigbe P, Tsui A (2016). Women’s fertility desires and contraceptive behavior in three peri-urban communities in sub Saharan Africa. Reprod Health.

